# Positive effects of a novel non-peptidyl low molecular weight radical scavenger in renal ischemia/reperfusion: a preliminary report

**DOI:** 10.1186/2193-1801-3-158

**Published:** 2014-03-24

**Authors:** Roberto Bassi, Andrea Vergani, Francesca D’Addio, Moufida Ben Nasr, Alessio Mocci, Maria Pia Rastaldi, Takaharu Ichimura, Joseph Vincent Bonventre, Paolo Fiorina

**Affiliations:** Nephrology Division, Boston Children’s Hospital, Harvard Medical School, Boston, MA USA; DiSTeBA, Universita’ del Salento, Lecce, Italy; Medicine, San Raffaele Scientific Institute, Milan, Italy; Department of Accident and Emergency, ASL, Bologna, Italy; Renal Research Laboratory, Fondazione IRCCS Ospedale Maggiore Policlinico & Fondazione D’Amico per la Ricerca sulle Malattie Renali, Milan, Italy; Renal Division, Brigham and Women’s Hospital, Harvard Medical School, Boston, MA USA

**Keywords:** Ischemia/reperfusion, Kidney disease, Kidney transplantation, Radical oxygen species, Inflammation

## Abstract

Ischemia/reperfusion (I/R) is one of the most common causes of acute kidney injury. Reactive oxygen species have been recognized to be an important contributor to the pathogenesis of I/R injury. We hypothesize that a non-peptidyl low molecular weight radical scavenger (IAC) therapy may counteract this factor, ultimately providing some protection after acute phase renal I/R injury. The aim of this preliminary study was to assess the ability of IAC to reduce acute kidney injury in C57BL/6 mice after 30-minute of bilateral ischemia followed by reperfusion. The rise in serum creatinine level was higher in C57BL/6 control mice after I/R when compared to IAC (1 mg)-treated mice. Control mice showed greater body weight loss compared to IAC-treated mice, and at pathology, reduced signs of tubular necrosis were also evident in IAC-treated mice. These preliminary evidences lay the basis for more comprehensive studies on the positive effects of IAC as a complementary therapeutic approach for acute phase renal I/R injury.

## Introduction

Kidney global or regional ischemia/reperfusion (I/R) is one of the most common causes of acute kidney injury (McCord 1985). During the peritransplant period, kidney transplanted patients are prone in 2-7% of the cases to experience I/R (Bonventre and Yang 2011), which can render the allograft more likely to develop acute rejection, and to progress towards long-term chronic allograft nephropathy (Thadhani et al. 1996; Cavaille-Coll et al. 2013). I/R injury is also a common event in a variety of pathological conditions such as diabetes and cardiovascular diseases (Luitse et al. 2012). Tissue hypoperfusion/hypoxia leads to depletion of cellular ATP and cytoskeleton damage (Singh et al. 2013). The restoration of blood flow with production of reactive oxygen species (ROS), and activation of leukocytes and endothelial cells (Rabb 2012; Ko et al. 2011) contribute to reperfusion injury. Although many experimental studies show a decreased injury and preserved renal function after dampening ROS production, efficient treatments are still limited (Cavaille-Coll et al. 2013; Leung et al. 2013; Venturini et al. 2006; Fiorina et al. 2006). Currently, the therapy for I/R injury is mainly based on supportive care and fluid administration (Cavaille-Coll et al. 2013; Luitse et al. 2012; Fiorina et al. 2005, 2006) and I/R injury remains a major cause of morbidity and mortality (Cavaille-Coll et al. 2013; Luitse et al. 2012; Fiorina et al. 2005, 2006). Non-peptidyl low molecular weight radical scavenger (IAC), a clinically available drug (D’Aleo et al. 2009), has been shown to have anti-oxidant properties in different models of brain and islet induced ischemia (D’Aleo et al. 2009; Corsi et al. 2011). We studied the effect of a IAC-based therapy in a murine model of bilateral kidney I/R injury, aiming to establish a proof-of-concept for the use of IAC as novel complementary therapy for individuals at risk for renal acute ischemic injuries.

## Materials and methods

### Animals

C57BL/6 (H-2Kb) mice were obtained from Jackson Laboratory (Bar Harbor, ME) and were housed in a pathogen-free environment; water and chow diet were provided ad libitum. Control (CTRL) and IAC-treated mice were weight (~20 grams), sex (male) and age (10 weeks-old) matched. Mice were cared for in accordance with institutional guidelines at the Harvard Medical School Facilities for Animal Care and Housing. Protocols were approved by the Harvard Animal Care and Use Committee.

### Interventional and functional studies

Two groups of C57BL/6 mice (n = 10 each) were subjected to experimental kidney ischemia through bilateral simultaneous clamping of vascular pedicles for 30 minutes, followed by clamp removal to allow kidney reperfusion. Mice were then treated with a single intraperitoneal injection of IAC (1 mg) or saline solution at 60 minutes after ischemic injury induction (30 minutes after clamps removal). Blood samples were collected by retro-orbital vein puncture before kidney ischemia induction (baseline; BL) at day (D)1, D2 and D4. Renal function was assessed by serum creatinine measurement by Creatinine Reagent Kit (Pointe Scientific, Lincoln Park, MI). Mouse weight was measured using a Pesola Digital Platform Scale (Pesola AG, Baar, Switzerland). IAC was kindly provided by Medestea Research and Production (Turin, Italy).

### Murine kidney pathology

Bilateral nephrectomy was performed at D4 in three mice per group for histological evaluation and acute tubular necrosis score computation (Fiorina et al. 2006). Kidney tissue was placed in 10% buffered formalin followed by paraffin embedding for haematoxylin and eosin staining. Histological slides for renal tissue damage evaluation, were examined by the operator without knowledge of the experimental design.

### Statistical analysis

Data are expressed as mean ± SD. Unpaired t-test was used to compare difference between groups. Statistical significance was set as p value < 0.05. Analysis of data was performed using STATA v12 statistical package for Windows (StataCorp, TX, USA).

## Results

Our preliminary results show that treatment with a single dose of IAC (1 mg) reduces kidney injuries in C57BL/6 mice during the first two days (acute phase) of experimentally induced bilateral I/R injury. In the untreated group (CTRL), one animal died after 48 hours, while none died in the IAC-treated group (p = ns, data not shown). The mouse from the CTRL group that did not survive, showed the highest serum creatinine value (2.60 mg/dl at D1). We intentionally used an interventional protocol for I/R with a low mortality rate, to gain better insight on the effect of IAC on kidney function rather than on a survival effect.

**Figure 1:**
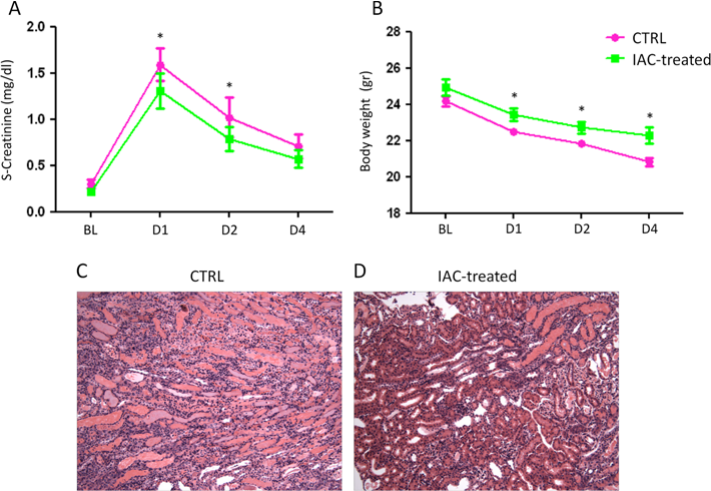
**IAC treatment partially prevented serum creatinine levels increase, body weight loss and acute tubular necrosis after I/R induction. (A)** Untreated control (CTRL) mice showed at day (D) 1 and D2 a more significant increase in serum creatinine levels after the induction of ischemia/reperfusion compared to non-peptidyl low molecular weight radical scavenger (IAC)-treated mice (D1 and D2: CTRL vs. IAC-treated, *p < 0.05). **(B)** Untreated CTRL mice showed a progressive and more evident reduction of body weight after ischemia/reperfusion induction compared to IAC-treated mice (D1, D2 and D4: CTRL vs. IAC-treated, *p < 0.05). **(C)** Untreated CTRL mice showed acute tubular necrosis signs in kidneys outer medulla with hyaline and granular casts accumulation. **(D)** Conversely, the extent of acute tubular necrosis was reduced in IAC-treated mice.

At baseline, serum creatinine levels were similar between CTRL and IAC-treated mice (BL: CTRL = 0.30 ± 0.40 vs. IAC-treated = 0.22 ± 0.30 mg/dl, p = ns), however a rapid increase in serum creatinine was observed in both groups soon after kidney I/R (Figure 1A). Serum creatinine levels in CTRL mice were significantly higher at D1 and D2 when compared to levels observed in IAC (1 mg)-treated mice (D1: CTRL = 1.59 ± 0.17 vs. IAC-treated = 1.31 ± 0.19 mg/dl, p = 0.02; D2: CTRL = 1.02 ± 0.21 vs. IAC-treated = 0.79 ± 0.13 mg/dl, p = 0.01), (Figure 1A). At D4 serum creatinine levels showed no difference between CTRL and IAC-treated mice, (D4: CTRL = 0.71 ± 0.13 vs. IAC-treated = 0.57 ± 0.10 mg/dl, p = ns), (Figure 1A).

Body weight was comparable between CTRL and IAC-treated mice at baseline (BL: CTRL = 24.1 ± 0.2 vs. IAC-treated = 24.9 ± 0.4 gr, p = ns), (Figure 1B). CTRL mice showed a progressive and more significant reduction of body mass during the follow-up period as compared to IAC-treated mice (D1: CTRL = 22.5 ± 0.2 vs. IAC-treated = 23.4 ± 0.3 gr, p = 0.02; D2: CTRL = 21.8 ± 0.1 vs. IAC-treated = 22.7 ± 0.3 gr, p = 0.02; D4: CTRL = 20.8 ± 0.2 vs. IAC-treated = 22.2 ± 0.4 gr, p = 0.01), (Figure 1B).

We finally examined the extent of kidney damage at D4 post I/R induction, particularly acute tubular necrosis (a hallmark of I/R injury). In CTRL mice, acute tubular necrosis was preferentially localized to the outer medulla of kidneys, with evident amounts of hyaline and granular casts (Figure 1C). In contrast, at D4 after I/R induction, in IAC-treated mice a marked reduction of acute tubular necrosis was evident as compared to untreated CTRL mice (Figure 1D). No signs of acute tubular necrosis were detectable in kidneys of sham-operated animals (data not shown).

## Discussion

I/R injury is an important contributor in acute kidney injury (Thadhani et al. 1996). After the ischemic event, organ reperfusion is accompanied by a cascade of inflammatory responses boosted by the local recruitment of peripheral leukocytes and the release of ROS (Rabb 2012). Together they lead to a common downstream pathway that results in the activation of pro-apoptotic genes such as caspase-3 and ultimately to acute kidney injury (Yang et al. 2005). Moreover, oxidative stress, occurring when ROS generation exceeds the capacity of anti-oxidant defenses, may cause indiscriminate damage to lipids, proteins and DNA, leading to future cell dysfunction and tissue damage (Yang et al. 2005). We here showed that non-peptidyl low molecular weight radical scavenger treatment, prevented serum creatinine increase, body weight loss and kidney tubular damage in C57BL/6 mice in the first two days after experimental kidney I/R injury. Thus, our preliminary results suggest that a non-peptidyl low molecular weight radical scavenger, having anti-oxidant properties, may warrant consideration as a complementary therapeutic strategy for the treatment of acute phase renal I/R injury in kidney transplanted patients (possibly reducing the risk for immediate acute rejection after transplant) or in individuals at risk for oxidative stress-related organ/tissue damage.

## Abbreviations

(S): Serum
(BL): Baseline.
